# Rheological and Quality Characteristics of Taftoon Bread as Affected by Salep and Persian Gums

**DOI:** 10.1155/2014/813286

**Published:** 2014-01-23

**Authors:** M. A. Sahari, R. Mohammadi, Z. Hamidi Esfehani

**Affiliations:** Food Technology Department, Faculty of Agriculture, Tarbiat Modares University, P.O. Box 14115-111, Tehran, Iran

## Abstract

Effects of salep gum at concentrations of 0.5%, 1%, 3%, and 5% (w/w flour basis) and the Persian gum at concentrations of 0.5%, 1%, and 3% (w/w flour basis) and combination of the two gums at concentrations of 0.5% + 0.5%, 0.75% + 0.25%, and 0.25% + 0.75% on rheological properties of the wheat flour dough and quality of Taftoon bread were studied with regard to retardation of staling. Rheological (farinograph and extensograph) characteristics, staling, and organoleptic evaluations were performed on the dough and the resulting Taftoon bread. Statistical results showed that the salep gum at 5% and Persian gum at 3% (w/w flour basis) had a significant effect on the dough properties. Salep and Persian gums when each separately added increased and decreased dough water absorption, respectively. Both hydrocolloids increased the dough resistance to extension and decreased its extensibility. Persian gum shows dual nature in water absorption and some other baking properties. Textural studies revealed that addition of 5% salep gum (w/w flour basis) reduced the bread crumb firmness and delayed the staling process of the Taftoon bread. X-ray diffraction study also confirmed this result.

## 1. Introduction

Bread, especially wheat bread, is widely consumed all over the world particularly in the developing countries and is an important source of protein and calories [1]. Bread is not spoiled by the microorganism or endogenous enzymes; however, its quality is lost by staling during storage [2]. Bread staling is a very complex process and many physicochemical changes occur that culminate in recrystallization of amylose and amylopectin [3]. There have been many approaches on how to retard the process, among which the most successful ones are the use of additives such as emulsifiers and enzymes [2]. There are several studies showing the potential use of hydrocolloids in baking industry as a way to retard staling [4–6].

Taftoon is one of the popular Iranian flat breads, produced from soft white wheat flours of higher extraction level. The bread has a round or oval shape, with the dimensions of 400–500 mm length, 400–500 mm width, and 2 mm thickness, with the average weight of 149-150 g. Ingredients of Taftoon bread are flour = 100 kg, water = 78–90 L, yeast = 450–650 g, and salt = 90–130 g. The chemical compositions of a loaf of Taftoon bread are crude protein = 10.47% dry mass, crude fat = 0.57% dries mass, crude fiber = 0.92% dry mass, ash = 2.53% crud mass, hydrocarbons = 85.5% dry mass, NaCl = 0.86% dry mass, and moisture = 26.87%, with the pH value of 7 and 389 kcal/100 energy [7].

Salep gum is produced by grinding the dried tubers of certain wild orchids; it is commonly used as a thickening and stabilizing agent; especially in ice-cream and desserts [8]. Salep is a valuable source for glucomannan [9].

Persian gum (zedu) is derived from *Amygdalus scoparia Spach*. Zedu is widely found in Iran, Iraq, Turkey, and Afghanistan. Chemical structure of zedu has not yet been studied in detail and is just known to contain a compound named cerasin. The substance is insoluble in cold water, while it shows properties similar to Arabic gum in boiling water [10].

As it has been shown, hydrocolloids can improve the quality of bakery products. Therefore, the aim of present study is to analyze the effects of salep and Persian gums on rheological and qualitative characteristics and retarding staling of Taftoon bread. To the best of our knowledge, this is the first study of this type on Taftoon bread.

## 2. Materials and Methods

### 2.1. Materials

Commercial wheat flour (87% extraction levels) containing 12.85% protein and 1.03% ash was obtained from a local market. Hydrocolloids in powder form were bought from a local market (salep gum: moisture = 10.83%, protein = 4.95%, ash = 1.91%, glucomannan = 51.2%, starch = 7.99%, and pH = 5.61; Persian gum: moisture = 8.89%, protein = 0.209%, ash = 2.48%, uronic acid = 9.70%, galactose = 28.20%, and pH = 4.62). Baker's yeast was obtained from the Fariman Company (Tehran, Iran). All chemical reagents were of analytical grade with the highest purity available.

Some characteristics of the flour such as protein, moisture, ash, and wet gluten content, as well as Zeleny sedimentation value and falling number, were determined according to the AACC standards [11]. The effect of hydrocolloids on dough mixing properties was determined by Brabender farinograph and extensograph (Brabender 1600, Sweden) according to the AACC guidelines [11].

### 2.2. Baking Process

Basic bread formula (traditional Taftoon bread) based on 1000 g flour consisted of compressed yeast 0.5%, salt 1%, and water up to consistency of 500 BU. Then, according to previous studies and our initial experiments, Persian gum was added to the flour at three concentration levels (W/W) of 0.5%, 1%, and 3%. Salep gum was also added at four concentrations levels (W/W) of 0.5%, 1%, 3%, and 5%. Moreover, a mixture of the two hydrocolloids at concentrations of 0.5% + 0.5%, 0.25% + 0.75%, and 0.75% + 0.25% were added to three samples. In this regard, the samples were as follows: Con: control without any gums, P(0.5): dough with 0.5% Persian gum, P(1): dough with 1% Persian gum, P(3): dough with 3% Persian gum, S(0.5): dough with 0.5% salep, S(1): dough with 1% salep, S(3): dough with 3% salep, S(5): dough with 5% salep, S(0.5)P(0.5): dough with 0.5% salep and 0.5% Persian gum, S(0.75)P(0.25): dough with 0.75% salep and 0.25% Persian gum, and P(0.75)S(0.25): dough with 0.75% Persian gum and 0.25% salep. The hydrocolloid levels were selected according to previous studies [1]. A straight dough process was carried out for preparing the bread. The ingredients were mixed and dough was fermented at 30°C for one hour, divided into 300 g pieces and proofed at 30°C for 15 min, and then sheeted and baked in a traditional oven at 200°C. Breads were cooled down at the room temperature and then packed into polyethylene packages.

### 2.3. Evaluation of Bread Quality

As bread staling is proportional to crumb firmness, crumb texture was assessed by a Texture Analyzer (Stable Microsystems, Surry, UK) regulated for bread under the following conditions: load cell, 1500 N; plunger speed, 120 mm/min; five-blade Kramer shear cell. Breads were prepared in small pieces (52∗52∗2 mm) for this study.

### 2.4. X-Ray Diffraction

For this study, Taftoon bread was prepared in small pieces (20∗20∗2 mm) and was placed in X-ray diffractometer after 0, 24, and 48 h storage at the room temperature [12]. X-ray patterns of bread were taken with Philips Analytical Diffractometer (Almelo, Netherland); type XPert MPD; with the generator setting of 40 kV, 30 mA; 2*θ* was 2–50° with a scanning speed of 0.02°/s and time per step of 1°; step size [°2*θ*]: 0.0200; and anode material of Co (*λ* = 1.78°A). The equipment works with Origin 75 and Microsoft Excel software.

### 2.5. Sensory Evaluation

The overall sensory score was taken by 30 semitrained panelists. Different sensory indexes such as shape, upper and lower surface characteristics, softness or hardness, and aroma were scored from 1 to 5 for poor to excellent, respectively [13]. According to Rajabzadeh [13], the following formula was used to calculate the overall score (*Q*).

*Q* = ∑(*P* · *G*)/(∑*G*) Where *P* is the sensory score and *G* is the coefficient of each sensory index.

*G* values for appearance, upper face properties, lower face properties, porosity, texture (firmness and softness), chewability, and aroma and flavor were 1, 2, 1, 2, 3, 2, and 9, respectively (suggested by Rajabzadeh) [13]. Evaluation of Iranian traditional breads is designed through the review and the comments received from the public. That based on scientific and qualitative criteria, empirical coefficients and according to the characteristics of each of the bread is done [13].

### 2.6. Statistical Analysis

All the chemical properties, dough rheology, and bread quality tests were performed in three replicates. The data were statistically analyzed by the analysis of variance in randomized complete blocks using the SAS software with eleven samples (including the control). The least significant difference (LSD) among samples was calculated at the significance level of 0.01.

## 3. Results and Discussion

### 3.1. Quality Characteristics in Wheat Flour

The wheat flour characteristics are shown in Table 1. Among the chemical characteristics, quantity and quality of protein was found to be the best index in predicting the quality of optimal breads. Results showed that the protein content was in the range of hard wheat. Breads of optimal quality are produced with flour from hard wheat's with protein contents of 10–12% [14].

### 3.2. Effect of Hydrocolloids on Farinograph Parameters

The effect of hydrocolloid addition on the farinograph measurements is summarized in Table 2. According to the table, water absorption by addition of salep and Persian gums has different trends. Various factors such as protein, starch, and sugar contents influence water absorption [15]. The highest absorption was observed when salep gum at the concentration of 5% was added and lowest adsorption was observed with addition of Persian gum at the concentration of 3%. The effect observed with salep gum agrees with increased water absorption found by Rosell et al. [5], when they added other hydrocolloids to the dough [5]. The effect is due to the presence of hydroxyl groups in the hydrocolloids structure [5, 16]. The effect of Persian gum on water absorption, however, disagrees with other studies on hydrocolloids. The water absorption of the flour depends on the swelling substances in the wheat (proteins and pentosans) and the mechanically damaged starch granules [17]. It seems that the higher value of protein in salep gum could explain its greater water absorption ability. The reason so far is unknown; it may be due to the presence of a substitute named “cerasin.” Cerasin is insoluble in cold water [10]. Addition of the hydrocolloid reduces the stability time, while increases the stability time [18]. This finding holds true in the Persian gum, but there are no specific trend in salep.

The highest degree of softening 10 min (indicating fast gluten network formation) after addition was observed for 0.75% Persian plus 0.25% salep gums and the lowest was observed for 3% Persian gum. The highest degree of softening 12 min (indicating good workability of the dough (strong dough)) after addition was observed for 0.5% and 1% Persian gum, while the lowest was observed for 5% salep gum. According to Pomeranz et al.'s [19] research, with increasing the softening degree, dough development time significantly reduced [19]. The degree of softening 10 min after addition confirms this result.

Each hydrocolloid affected the time required to reach dough development time (consistency) of 500 BU in a different manner. The longest development time was observed with salep gum at the concentration of 5% and the shortest time was observed for 0.75% salep gum plus 0.25% Persian gum. Persian gum at 3% concentration and salep gum at 5% concentration increased the stability significantly, and the control and other samples were not significantly different in this regard.

Findings of the present study showed no specific trend of stability in the salep and Persian gums. Various gums have different behaviors in relation to the duration of the show dough [5, 18]. It seems that the different behaviors come from the differences in the structure and chemical compositions of the gums.

### 3.3. Effect of Different Hydrocolloids on the Extensograph Parameters

The effects of adding hydrocolloids on the extensograph measurements after resting time of 45, 90, and 135 minutes are shown in Tables 3, 4, and 5, respectively. Extensograph measures of dough extensibility and resistance to extension, and thus the viscoelastic behavior of the dough, can be determined. The energy or work input required for the deformation increased by addition of salep and Persian gums. Dough containing 3% Persian gum showed the highest energy. Moreover, at each time measured (after 45, 90, and 135 min), control sample showed the lowest energy. The effect observed agrees with increased energy found by Guarda et al. [18], when they added xanthan and K-carrageenan to the dough [18].

The control sample exhibited higher extensibility and dough containing hydrocolloids exhibited lower extensibility after 45, 90, and 135 minutes. Samples of 5% salep gum and 0.5% Persian gum separately showed the lowest extensibility. This is inconsistent with the results reported by Rosell et al. [5]. It seems that by adding these two gums, the dough becomes too strong.

By adding the hydrocolloids, resistance to extension and the ratio of resistance to extension or extensibility increased. Salep gum and Persian gum at concentrations of 5% and 3%, respectively, showed the highest and the control sample showed the lowest values of resistance to extension and resistance to extension ratio after the resting time of 45, 90, and 135 minutes. The results obtained in the current study showed that by adding these two gums, dough are stronger. This is in agreement with the findings of Tavakolipour and Kalbasi-Ashtari [1] and Guarda et al. [18].

The highest farinograph quality value was obtained for addition of 3% Persian gum and addition of 1% salep gums, while the lowest value was observed for addition of 0.5% Persian gum and also addition of 0.75% Persian gum and 0.25% salep gum. The farinograph quality values (Valorimeter value) were obtained according to development time and stability. As it was shown, when the quality value was higher, the dough rheological properties were better [14].

### 3.4. Effect of Hydrocolloids on Quality of Bread Texture

With regard to the farinograph and extensograph results, significant differences were observed between the control sample and the sample containing 5% salep plus 3% Persian gums. Therefore, bread staling and quality can be assayed using these three samples. Shear force values of breads during 48 h of storage are summarized in Table 6. The results showed that when the staling of bread occurs, texture of bread becomes stiffer. The effect observed is in agreement with increased shear force found by Tavakolipour and Kalbasi-Ashtari [1], when they added hydroxy propyl methylcellulose (HPMC) and carboxy methyl cellulose (CMC) to the dough [1]. However, this is inconsistent with the results reported by Rosell et al. [5] when they added xanthan to the dough. Salep gum at 5% and Persian gum at 3% concentrations significantly decreased and increased the shear force, respectively (*P* < 0.01). Decreased shear force value in bread containing 5% salep gum is probably due to the high water absorption ability of this hydrocolloid. Shear force value after 48 h for the bread containing 5% salep gum is lower than that for the control bread immediately after baking. So, salep gum acts as antistaling agent, while Persian gum at any time increases the shear force value. It seems that the greater water absorption ability in salep gum could explain the antistaling act. This factor can delay the crystallization of starch [1, 4, 5].

### 3.5. Effect of Hydrocolloids on Sensory Properties of Breads

Effect of hydrocolloids on the sensory parameters is presented in Table 7. Among different sensory parameters, “firmness and softness” of the breads containing hydrocolloids showed significantly higher scores (*P* < 0.01). With regard to the firmness and softness parameters, our results are in agreement with the findings of Tavakolipour and Kalbasi-Ashtari [1]. Bread containing 3% Persian gum had the highest score in appearance properties. The samples were not significantly different in overall score, including the scores of aroma and flavor, porosity, and lower face properties.

### 3.6. X-Ray Diffraction Results

**Figure 1:**
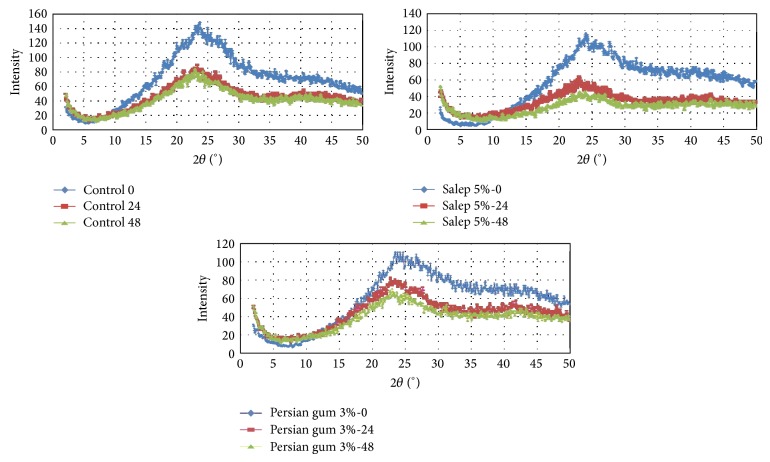
The X-ray diffraction diagrams of fresh and aged Taftoon bread after storage time of 0, 24, and 48 h in the control sample (bread without any gums), bread containing 5% salep gum, and bread containing 3% Persian gum.

Figure 1 shows the X-ray diffraction diagrams of fresh and aged Taftoon bread after the storage time of 0, 24, and 48 h in the control (bread without any gums), bread with 3% Persian gum, and bread with 5% salep gum, respectively. It is noticeable that peak intensities, that is, starch crystallinity, increased with time and the peak areas in bread samples were 3365.955, 2201.876, and 2039.580 cm^2^ (in the control); 2761.297, 2101782, and 1836.068 cm^2^ (in the sample containing 3% Persian gum); 2728.964, 1616.006, and 1325.967 cm^2^ (in the sample containing 5% salep gum) after 0, 24, and 48 h, respectively. Results showed that the decrease in the peak area in the bread sample containing 5% salep gum was higher (291 cm^2^), when compared with the bread sample containing 3% Persian gum (265 cm^2^) and the control sample (162 cm^2^). Furthermore, salep gum (at 5% w/w flour basis) acted as an antistaling agent due to the high water absorption ability of this hydrocolloid [20].

## 4. Conclusion

This study shows that salep gum and Persian gum increased and decreased water absorption of dough, respectively. The farinograph quality value obtained shows that Persian gum decreased the water absorption, while improved some other baking properties of dough and so shows dual nature of Persian gum. Addition of hydrocolloids increased the energy required for deformation and decreased the extensibility. Addition of salep gum at the concentration of 5% prolongs freshness of bread during storage at the room temperature. This was confirmed by X-ray diffraction studies. Sensory assay revealed that hydrocolloid significantly increases the softness of bread, while no statistically significant changes occurred in the overall palatability.

## Figures and Tables

**Table 1:** Flour properties.

	Wet gluten, %	Falling number	Zeleny value, mL	Total ash, %	Moisture, %	Protein, %
Flour	25.6 ± 0	404.5 ± 5.5	21 ± 0	1.03 ± 0.01	11.5 ± 0	12.85 ± 0.03

Values are the mean ± standard deviation (*n* = 3).

**Table 2:** Effect of hydrocolloids on farinograph parameters.

Sample	Water absorption, %	Development time, min	Degree of softening (12 min after max), Brabender unit	Degree of softening (10 min after begin), Brabender unit	Stability, min	Farinograph quality number
Con	63.00 ± 0^ cdef^	5.25 ± 0.50^c^	75.50 ± 1.50^ab^	39.50 ± 0.50^abc^	6.30 ± 0^c^	88.00 ± 2.00^bc^
P(0.5)	62.00 ± 0^fg^	5.15 ± 0.15^cd^	84.00 ± 12.00^a^	48.00 ± 11.00^ab^	5.60 ± 0.80^c^	79.50 ± 7.50^c^
P(1)	61.66 ± 0^g^	5.10 ± 0.10^cd^	78.00 ± 15.00^ab^	48.00 ± 9.00^ab^	6.45 ± 0.55^c^	82.50 ± 5.50^bc^
P(3)	58.33 ± 0^h^	6.65 ± 1.15^b^	50.00 ± 2.00^c^	18.50 ± 4.50^d^	11.00 ± 0.40^a^	124.00 ± 11.00^a^
S(0.5)	63.66 ± 0^cd^	5.25 ± 0.45^c^	64.50 ± 5.50^abc^	34 ± 6.00^abcd^	6.65 ± 0.75^c^	94.50 ± 8.50^bc^
S(1)	64.00 ± 0^c^	3.80 ± 0.50^ef^	57.50 ± 12.5^abc^	31.50 ± 10.50^bdc^	6.90 ± 1.70^c^	103.50 ± 17.50^ab^
S(3)	68.33 ± 0^b^	3.95 ± 0.25^def^	65.00 ± 9.00^abc^	39.00 ± 8.00^abc^	5.85 ± 0.25^c^	87.50 ± 8.50^bc^
S(5)	73.00 ± 0^a^	8.20 ± 0^a^	0.0^d^	25.50 ± 6.50^cd^	8.90 ± 0.40^b^	99.00 ± 12.00^bc^
S(0.5)P(0.5)	62.66 ± 0^defg^	4.95 ± 0.05^cde^	67.00 ± 11.00^abc^	38.50 ± 8.50^abc^	6.90 ± 0.70^c^	90.00 ± 9.00^bc^
S(0.75)P(0.25)	63.33 ± 0^cde^	3.55 ± 1.25^f^	67.50 ± 5.50^abc^	41.00 ± 9.00^abc^	6.20 ± 0.80^c^	87.00 ± 11.00^bc^
P(0.75)S(0.25)	62.33 ± 0^efg^	4.55 ± 0.25^cdef^	75.50 ± 10.50^ab^	49.50 ± 6.50^a^	6.20 ± 0.80^c^	79.00 ± 4.00^c^

Con: control without any gums, P(0.5): dough with 0.5% Persian gum, P(1): dough with 1% Persian gum, P(3): dough with 3% Persian gum, S(0.5): dough with 0.5% salep, S(1): dough with 1% salep, S(3): dough with 3% salep, S(5): dough with 5% salep, S(0.5)P(0.5): dough with 0.5% salep and 0.5% Persian gum, S(0.75)P(0.25): dough with 0.75% salep and 0.25% Persian gum, and P(0.75)S(0.25): dough with 0.75% Persian gum and 0.25% salep. Values are the mean ± standard deviation (*n* = 3). Values represented by different letters in each column are significantly different (*P* < 0.01).

**Table 3:** Effect of hydrocolloids on extensograph parameters after 45 min resting time.

Samples	Dough energy (cm^2^)	Extensibility (mm)	Resistance to extension (BU)	Resistance to extension/extensibility
Con	35.50 ± 2.50^e^	151.50 ± 9.50^a^	148.00 ± 2.00^h^	0.95 ± 0.05^g^
P(0.5)	37.00 ± 2.00^de^	108.00 ± 1.00^ef^	235.00 ± 14.00^d^	2.15 ± 0.15^c^
P(1)	49.50 ± 3.50^c^	139.00 ± 9.00^abc^	230.00 ± 3.00^de^	1.65 ± 0.05^def^
P(3)	71.00 ± 4.00^a^	127.00 ± 1.00^cd^	360.50 ± 12.50^b^	2.85 ± 0.05^b^
S(0.5)	40.00 ± 0^de^	143.30 ± 3.46^ab^	186.50 ± 3.50^g^	1.35 ± 0.05^f^
S(1)	48.00 ± 1.00^c^	134.00 ± 4.00^c^	239.50 ± 8.50^d^	1.80 ± 0.10^cde^
S(3)	57.00 ± 2.00^b^	109.50 ± 5.50^ef^	338.00 ± 5.00^c^	3.10 ± 0.20^b^
S(5)	58.00 ± 3. 00^b^	97.00 ± 9.00^f^	391.50 ± 9.50^a^	4.05 ± 0.45^a^
S(0.5)P(0.5)	41.50 ± 2.50^d^	137.50 ± 6.50^bc^	197.50 ± 6.50^fg^	1.45 ± 0.05^ef^
S(0.75)P(0.25)	47.50 ± 0.50^c^	133.50 ± 4.5^bc^	237.00 ± 5.00^d^	1.80 ± 0^cde^
P(0.75)S(0.25)	38.00 ± 1.00^de^	114.00 ± 3.00^de^	213.00 ± 1.00^ef^	1.85 ± 0.05^cd^

Con: control without any gums, P(0.5): dough with 0.5% Persian gum, P(1): dough with 1% Persian gum, P(3): dough with 3% Persian gum, S(0.5): dough with 0.5% salep, S(1): dough with 1% salep, S(3): dough with 3% salep, S(5): dough with 5% salep, S(0.5)P(0.5): dough with 0.5% salep and 0.5% Persian gum, S(0.75)P(0.25): dough with 0.75% salep and 0.25% Persian gum, and P(0.75)S(0.25): dough with 0.75% Persian gum and 0.25% salep. Values are the mean ± standard deviation (*n* = 3). Values with different letters in each column are significantly different (*P* < 0.01).

**Table 4:** Effect of hydrocolloids on extensograph parameters after 90 min resting time.

Samples	Dough energy (cm^2^)	Extensibility (mm)	Resistance to extension (BU)	Resistance to extension/extensibility
Con	27.00 ± 0^f^	141.50 ± 7.50^a^	125.00 ± 6.00^h^	0.90 ± 0.10^h^
P(0.5)	36.00 ± 0^de^	95.00 ± 0^f^	271.00 ± 4.00^c^	2.85 ± 0.05^c^
P(1)	39.00 ± 3.00^cd^	127.50 ± 4.50^bcd^	205.50 ± 5.50^e^	1.60 ± 0^ef^
P(3)	66.50 ± 0.50^a^	116.00 ± 0d^e^	391.50 ± 0.50^a^	3.40 ± 0^a^
S(0.5)	30.00 ± 0^f^	132.00 ± 0^abc^	150.00 ± 0^g^	1.10 ± 0^g^
S(1)	35.0 ± 3^de^	125.50 ± 12.50^bcd^	194.00 ± 3.00^e^	1.55 ± 0.15^f^
S(3)	50.00 ± 1.00^b^	120.50 ± 2.50^d^	274.00 ± 9.00^c^	2.30 ± 0.10^d^
S(5)	48.50 ± 0.50^b^	103.00 ± 2.00^ef^	326.50 ± 5.50^b^	3.20 ± 0^b^
S(0.5)P(0.5)	34.00 ± 2.50^e^	137.00 ± 10.00^ab^	169.00 ± 5.00^f^	1.25 ± 0.05^g^
S(0.75)P(0.25)	37.50 ± 2.50^cde^	132.50 ± 4.50^abc^	195.00 ± 4.00^e^	1.50 ± 0^f^
P(0.75)S(0.25)	40.50 ± 1.50^c^	124.5 ± 0.50^bcd^	219.50 ± 6.50^d^	1.75 ± 0.05^e^

Con: control without any gums, P(0.5): dough with 0.5% Persian gum, P(1): dough with 1% Persian gum, P(3): dough with 3% Persian gum, S(0.5): dough with 0.5% salep, S(1): dough with 1% salep, S(3): dough with 3% salep, S(5): dough with 5% salep, S(0.5)P(0.5): dough with 0.5% salep and 0.5% Persian gum, S(0.75)P(0.25): dough with 0.75% salep and 0.25% Persian gum, and P(0.75)S(0.25): dough with 0.75% Persian gum and 0.25% salep. Values are the mean ± standard deviation (*n* = 3). Values with different letters in each column are significantly different (*P* < 0.01).

**Table 5:** Effect of hydrocolloids on extensograph parameters after 135 min resting time.

Samples	Dough energy (cm^2^)	Extensibility (mm)	Resistance to extension (BU)	Resistance to extension/extensibility
Con	25.50 ± 0.50^g^	146.50 ± 1.50^a^	117.50 ± 2.50^g^	0.80 ± 0^e^
P(0.5)	39.50 ± 2.50^c^	94.00 ± 3.00^f^	303.50 ± 12.50^b^	3.25 ± 0.05^a^
P(1)	35.00 ± 0^de^	131.00 ± 0^bc^	171.50 ± 7.50^e^	1.30 ± 0.10^d^
P(3)	64.00 ± 1.00^a^	117.00 ± 1.00^e^	376.50 ± 3.50^a^	3.20 ± 0^a^
S(0.5)	25.50 ± 1.50^g^	122.50 ± 6.50^de^	138.00 ± 0^f^	1.15 ± 0.05^d^
S(1)	29.50 ± 3.50^f^	130.00 ± 2.00^c^	159.50 ± 6.50^e^	1.25 ± 0.05^d^
S(3)	45.00 ± 2.00^b^	133.00 ± 3.00^bc^	231.50 ± 2.50^c^	1.75 ± 0.05^c^
S(5)	44.00 ± 1.00^b^	99.50 ± 2.50^f^	295.50 ± 13.50^b^	3.00 ± 0.20^b^
S(0.5)P(0.5)	34.00 ± 1.00^de^	138.00 ± 4.00^b^	166.50 ± 2.50^e^	1.20 ± 0^d^
S(0.75)P(0.25)	32.00 ± 1.00^ef^	128.50 ± 2.50^cd^	167.50 ± 2.50^e^	1.30 ± 0^d^
P(0.75)S(0.25)	37.00 ± 1.00^cd^	119.50 ± 2.50^e^	208.00 ± 7.00^d^	1.70 ± 0.10^c^

Con: control without any gums, P(0.5): dough with 0.5% Persian gum, P(1): dough with 1% Persian gum, P(3): dough with 3% Persian gum, S(0.5): dough with 0.5% salep, S(1): dough with 1% salep, S(3): dough with 3% salep, S(5): dough with 5% salep, S(0.5)P(0.5): dough with 0.5% salep and 0.5% Persian gum, S(0.75)P(0.25): dough with 0.75% salep and 0.25% Persian gum, and P(0.75)S(0.25): dough with 0.75% Persian gum and 0.25% salep. Values are the mean ± standard deviation (*n* = 3). Values with different letters in each column are significantly different (*P* < 0.01).

**Table 6:** Shear force values of breads.

Samples	Shear force after 0 h of storage, *N*	Shear force after 24 h of storage, *N*	Shear force after 48 h of storage, *N*
Con	727.42 ± 50.17^b^	916.5 ± 6.5^b^	1136.87 ± 38.12^a^
P(3)	889.50 ± 2.50^a^	1140.00 ± 76.25^a^	1194.37 ± 26.87^a^
S(5)	562.50 ± 6.0^c^	630.37 ± 11.62^c^	655.87 ± 25.12^b^

Con: control without any gums, P(3): dough with 3% Persian gum, and S(5): dough with 5% salep. Values are the mean ± standard deviation (*n* = 3). Values with different letters in each column are significantly different (*P* < 0.01).

**Table 7:** The effect of hydrocolloids on the sensory parameters.

	Con	P(3)	S(5)
Appearance	4.30 ± 0.91^b^	4.80 ± 0.40^a^	3.85 ± 1.28^b^
Upper face properties	7.70 ± 2.03^ab^	8.50 ± 1.22^a^	6.60 ± 3.02^b^
Lower face properties	3.85 ± 1.36^a^	4.35 ± 0.64^a^	4.20 ± 0.88^a^
Porosity	6.40 ± 3.25^a^	7.70 ± 1.57^a^	6.40 ± 2.69^a^
Texture (firmness and softness)	9.00 ± 4.92^b^	10.95 ± 1.67^a^	11.55 ± 3.34^a^
Chewability	6.10 ± 3.59^b^	7.90 ± 1.47^a^	7.40 ± 2.41^ab^
Aroma and flavor	28.80 ± 15.20^a^	30.15 ± 11.22^a^	26.55 ± 13.01^a^
Overall score	3.30 ± 1.40^a^	3.71 ± 0.74^a^	3.30 ± 1.18^a^

Con: control without any gums, P(3): dough with 3% Persian gum, and S(5): dough with 5% salep gum. Values are the mean ± standard deviation (*n* = 3). Values with different letters in each row are significantly different (*P* < 0.01).
